# Adding ofloxacin to standard triple-drug regimens increased the Helicobacter pylori eradication rate: Data from randomized clinical trial 

**DOI:** 10.22088/cjim.8.2.108

**Published:** 2017

**Authors:** Mahmoud Sadeghi-Hadad-Zavareh, Seyyedsaeid Mohammadi, Mahmoud Hajiahmadi, Mostafa Javanian, Masoomeh Habibian

**Affiliations:** 1 Infectious Diseases and Tropical Medicine Research Center, Health Research Institute, Babol University of Medical Sciences, Babol, Iran.; 2 Cancer Research Center, Health Research Institute, Babol University of Medical Sciences, Babol, Iran.; 3 Non-Communicable Pediatric Diseases Research Center, Babol University of Medical Sciences, Babol, Iran.; 4 Student Research Committee, Babol University of Medical Sciences, Babol, Iran.

**Keywords:** Helicobacter pylori, Dyspepsia, Triple therapy, Ofloxacin

## Abstract

**Background:**

The rate of Helicobacter pylori (H.pylori) eradication in dyspeptic patients using bismuth- based triple therapy is low due to bacterial that are resistant to antibiotics. The results of recent studies regarding levofloxacin have been encouraging, but the high cost of treatment prevents its routine administration. We, therefore, performed the present double-blind clinical trial to compare the efficiency of quadruple-drug regimen containing ofloxacin, clarithromycin, amoxicillin, and omeprazole and the same standard triple-therapy regimen minus ofloxacin in H. pylori positive dyspepsia.

**Methods:**

The study patients were recruited among dyspeptic patients requiring gastroscopy. Patients with the history of H.pylori treatment, renal failure and pregnancy were excluded. Diagnosis of H.pylori infection was confirmed by rapid urease test and response to treatment was confirmed via negative urease breath test (UBT) 20 days after completion of treatment. Patients were allocated intermittently to standard triple therapy containing amoxicillin, clarithromycin, omeprazole alone or plus ofloxacin for ten days. Response to treatment was compared between the two groups.

**Results:**

A total of 140 patients entered the study (70 patients in each group). At endpoint 30 (42.9%) patients of group 1 and 39 (55.7%) patients of group 2 became asymptomatic. Furthermore, 55 (78.6%) patients of group 1 and 66 (94.3%) patients of group 2 revealed negative urea breath test (P= 0.01).

**Conclusion:**

This study indicates adding ofloxacin to standard triple-therapy for H.pylorri infection significantly increases the rate of eradication. These findings highlight ofloxacin as empiric treatment of H. pylori infection.

Helicobacter pylori (H. pylori) eradication is recommended in many conditions including peptic ulcer disease, MALToma, and gastric cancer (1-5). Empirical regimens are expected to eradicate HP infection in more than 90% of patients. Research in Iran and other Asian countries showed that about 50% of the H.pylori infection are resistant to metronidazole (6, 7) and 17% to clarithromycin. The inefficiency of bismuth-based therapy for H.pylori has been shown (8, 9) and so many researchers recommend quadruple-therapy using levofloxacin as their first line treatment (10, 11). However, because of high cost, ofloxacin is used instead of levofloxacin for H.pylori eradication in Iran. Nonetheless, the data regarding the efficiency of ofloxacin in H.pylori infection are scarce. Thus, the present study was conducted to determine the eradication rate of H.pylroi infection with quadruple regimen containing clarithromycin, amoxicillin, ofloxacin and omeprazole.

## Methods

**Figure 1:**
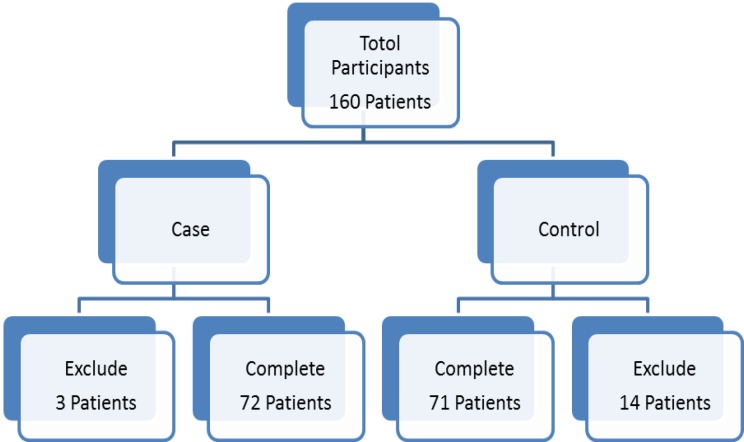
The exclusion flow chart of studied patient

This double- blind, randomized clinical trial was performed in Ayatollah Rouhani Hospital during 2014 to 2015 (IRCT2015113025292N1). The study patients were those required to have diagnostic upper endoscopy for dyspepsia. Exclusion criteria were history of previous treatment for H.pylori, presence of renal failure and pregnancy (fig 1). This study was approved by the Ethics Committee of Babol University of Medical Sciences. Informed consent was taken from all the study participants. All patients underwent endoscopy performed by a single gastroenterologist and the presence of H.pylori infection was confirmed with rapid urease test (RuT) using Heliprobe kit (Kibion, Uppsala, Sweden).

Drug regimens were similar and all participants provided at least one regular prescription medication. Patients were alternately allocated to receive either group A regimen containing amoxicillin (500 mg orally twice daily for 10 days, Farabi co. Iran), clarithromycin (500 mg, orally twice daily for 10 days, Farabi co, Iran), omeprazole (20 mg orally twice daily for 10 days, Abidi co, Iran) or group B with same standard regimen plus ofloxacin (200 mg orally twice daily for 5 days, Exir co, Iran). Response to treatment was considered as negative UBT which was performed 20 days after completion of treatment. In addition, the two groups were compared with regard to the frequency of gastrointestinal symptoms. Data collection regarding treatment response was provided by the gastroenterologist who was blind to treatment regimen. Statistical analysis was also performed by a blind analyzer. SPSS Version 20 and chi-square test were used for statistical analysis.

## Results

A total of 140 patients (70 participants for each group) entered the study. The baseline characteristics of the study population in both treatment groups are presented in table 1. Over the study period, 13 patients lost to follow-up and 3 patients did not complete treatment. At endpoint, 55 (78.6%) patients taking regimen A and 66 (94.3%) patients taking regimen B responded to treatment (P=0.01). Furthermore, at the end of the study period 30 (42.9%) patients of group A and 39 (55.7%) patients of group B became asymptomatic. Table 1 shows the baseline characteristics of all individuals enrolled.

**Table 1:** Basic characteristic of studied patients

**Variables**	**Control** **N (%)**	**Case** **N (%)**	**P-value**
**Sex**			
Male Female	28(40) 42(60)	37(52.9) 33(47.1)	0.175
**Age group**			
Less than 40 years More than 40 years	23(32.9) 47(67.1)	35(50) 35(50)	0.115
**Level of Education**			
Below high school Diploma Bachelor diploma and higher	9(12.9) 49(70) 12(17.1)	14(20) 45(64.3) 11(15.7)	0.245

After completion of treatment, 30 (42.9%) patients of group A and 39 (55.7%) patients of group B did not have any symptoms. 55 (78.6%) cases in group A and 66 (94.3%) cases in group B had negative UBT. Based on the comparison between two groups of drug regimen and the eradication rate of infection, there is a significant relation (P=0.01) (table 2).

**Table 2:** Univariate analysis of the clinical factors influencing the efficacy of H.pylori eradication

**Characteristics**	**Eradication rate (%)**	**P- value**
**Age**		
<40 ≥40	89.7 84.1	0.45
**Sex**		
Male Female	89.2 84	0.46
**Residency**		
Urban Rural	84.1 81.7	0.46
**Regimen**		
A* B**	78.6 94.3	0.01

* Amoxicillin, Clarithromycin, omeprazole

** A+ Ofloxacin

## Discussion

 The findings of this study revealed significantly the higher rate of H.pylori eradication at 93.5% in drug regimen containing ofloxacin. Regarding patients’ selection and study design, greater efficiency of quadruple therapy should be attributed to ofloxacin. Empirical therapy for H.pylori infection should be effective in more than 90% of patients, whereas in Iran, the efficacy of standard therapy is about 80% (10) which may be attributed to drug resistance, particularly bismuth or metronidazole (11). As observed in the present study, standard therapy was effective only in 78.6% of the treated patients. These results suggest to include ofloxacin in empirical treatment, in particular since ofloxacin compared with leveofloxacine is cheaper in price and makes it the preferred choice in empiric therapy.

This effect of treatment was not related to age, gender and location. In a study by Sierra et al., successful eradication of triple therapy was less than 80%. In the current study, H.pylori eradication triple therapy rate was 78.57% which was comparable to our study. The authors found no additional efficacy by including bismuth to triple therapy and recommended the levofloxacin triple therapy as the first-line therapy for H.pylori eradication (12). Irardi et al. found greater efficiency with triple therapy based on amoxicillin, clarithromycin and rabeprazole (7). In a study, H.pylori eradication rate was below 90%, which was consistent with the results reported by Federico et al. The authors recommended the use of floroquinolones as the first-line therapy for H.pylori eradication (6).

 In another study conducted by Onyekwere et al., the eradication rate of triple therapy including amoxicillin, clarithromycin and omeprazole was compared according to duration treatment period. In this study, the results of 7-day and 14-day treatment were comparable (13). 

In conclusion, the results of this study indicate that adding ofloxacin to triple-therapy regimen increases the rate of H.pyloryi eradication and highlights the quadruple regimen as the first line empiric therapy in dyspeptic patients. Due to its lower cost, easy accessibility and higher rate of treatment response, ofloxacin should be considered as the H.pylori infection treatment in Iran.
